# Multimodal Sensing-Enabled Large Language Models for Automated Emotional Regulation: A Review of Current Technologies, Opportunities, and Challenges

**DOI:** 10.3390/s25154763

**Published:** 2025-08-01

**Authors:** Liangyue Yu, Yao Ge, Shuja Ansari, Muhammad Imran, Wasim Ahmad

**Affiliations:** James Watt School of Engineering, University of Glasgow, Glasgow G12 8QQ, UK; 2522553y@student.gla.ac.uk (L.Y.); shuja.ansari@glasgow.ac.uk (S.A.); muhammad.imran@glasgow.ac.uk (M.I.); wasim.ahmad@glasgow.ac.uk (W.A.)

**Keywords:** multimodal sensing, large language model, emotional regulation

## Abstract

Emotion regulation is essential for mental health. However, many people ignore their own emotional regulation or are deterred by the high cost of psychological counseling, which poses significant challenges to making effective support widely available. This review systematically examines the convergence of multimodal sensing technologies and large language models (LLMs) for the development of Automated Emotional Regulation (AER) systems. The review draws upon a comprehensive analysis of the existing literature, encompassing research papers, technical reports, and relevant theoretical frameworks. Key findings indicate that multimodal sensing offers the potential for rich, contextualized data pertaining to emotional states, while LLMs provide improved capabilities for interpreting these inputs and generating nuanced, empathetic, and actionable regulatory responses. The integration of these technologies, including physiological sensors, behavioral tracking, and advanced LLM architectures, presents the improvement of application, moving AER beyond simpler, rule-based systems towards more adaptive, context-aware, and human-like interventions. Opportunities for personalized interventions, real-time support, and novel applications in mental healthcare and other domains are considerable. However, these prospects are counterbalanced by significant challenges and limitations. In summary, this review synthesizes current technological advancements, identifies substantial opportunities for innovation and application, and critically analyzes the multifaceted technical, ethical, and practical challenges inherent in this domain. It also concludes that while the integration of multimodal sensing and LLMs holds significant potential for AER, the field is nascent and requires concerted research efforts to realize its full capacity to enhance human well-being.

## 1. Introduction

Mental health issues have become common public health concerns worldwide, significantly influencing individual well-being, productivity, and overall life satisfaction. Globally, mental disorders such as anxiety, depression, and stress-related conditions are increasing dramatically, placing a substantial burden on healthcare systems and societies [1,2]. It is estimated that approximately eight million people lose their lives each year due to various mental illnesses and stress-related conditions [3,4,5]. One major challenge in addressing these issues is the limited availability and accessibility of qualified mental health professionals, especially in low-resource settings or for individuals facing logistical, financial, or cultural barriers to in-person support for emotional regulation. Compounding the issue, the societal stigma associated with mental illness often deters individuals from seeking necessary care, driven by fears of judgment or concerns about treatment costs and effectiveness [6]. The complexity and variability of these disorders mean that traditional methods relying solely on verbal communication can be insufficient for accurate diagnosis and effective intervention.

The exploration of automating emotional support originated from early innovations such as ELIZA [7] in 1966, a rule-based chatbot developed by Joseph Weizenbaum, which provided rudimentary therapeutic dialogues through simple text interactions. Decades later, more sophisticated approaches emerged, as exemplified by Woebot [8] in 2017, an AI chatbot leveraging cognitive behavioral therapy (CBT) to offer structured emotional interventions. Recently, advancements in large language models (LLMs), notably ChatGPT and GPT-4, significantly enhanced AI’s capacity for nuanced, empathetic interactions, paving the way for context-aware and personalized Automated Emotional Regulation (AER) systems [9].

In response to these escalating challenges, AER systems offer a promising solution to mental healthcare. These interventions leverage digital technologies, including mobile applications [10,11], websites [11,12], and virtual reality (VR) [13,14,15], which can provide continuous, personalized support, making mental health services more inclusive and responsive to individual needs. AER is broadly defined as therapeutic interventions delivered via digital technologies for improving mental health, treating symptoms, or managing mental health difficulties. Examples of automated systems include artificial intelligence (AI)-driven chatbots simulating therapeutic conversations, VR systems for exposure therapy or skills training, and even social robots designed to assist in specific therapeutic contexts, such as helping individuals with social anxiety rehearse social situations, alleviating depressive symptoms in geriatric care, or aiding children with autism spectrum disorder (ASD) in developing social skills [16]. These automated systems aim not only to address healthcare demands but also to offer intrinsic therapeutic benefits that complement existing approaches.

Effective emotional regulation, whether human-delivered or automated, often hinges on understanding the patient’s internal emotional state. This includes recognizing their emotions, gauging their level of engagement, and establishing therapeutic rapport. Traditional methods rely on clinician observation and patient self-report, which can be limited by biases and the inability to capture dynamic changes. In digital contexts, multimodal emotion recognition (MER) offers a potential solution for gaining deeper insights into a user’s emotional state [17,18]. MER involves capturing and analyzing data from multiple sources or modalities simultaneously, such as visual, auditory signals, textual content, and physiological responses, to identify and comprehend human emotional states. This approach acknowledges that human emotions are multifaceted, and they are conveyed through coordinated patterns across different channels. This allows for a more holistic understanding of an individual’s emotion and emotional state, overcoming the limitations of unimodal assessments.

Concurrently, the field of AI has witnessed the rapid rise in LLMs [19], which possess remarkable capabilities in processing, understanding, and generating human-like language. Mental health is considered a particularly suitable domain for LLMs because emotional regulation is fundamentally rooted in language [20]. LLMs are being explored for various applications, including analyzing clinical notes or patient-generated texts for diagnostic insights [21,22], summarizing clinical information [23], and constructing conversational agents [24,25,26] designed to provide support or deliver psychoeducation. The potential for LLMs to mimic empathy and adhere to therapeutic protocols offers possibilities for creating more engaging and potentially effective automated interventions.

This confluence of multimodal sensing technologies and the powerful reasoning and generative capabilities of LLM heralds a new frontier: the development of AER systems. This review holds the potential to overcome many limitations of previous AER attempts by enabling more context-aware and personalized emotional support. Such integrated systems could transform mental healthcare by facilitating early detection of emotional distress and delivering tailored interventions, as well as revolutionize human–computer interactions (HCIs) by fostering more empathetic and adaptive AI agents. A key potential lies in creating closed-loop AER systems where an LLM interprets multimodally sensed emotional states and generates adaptive regulatory responses, positioning the LLM as an active participant or “co-creator” in the user’s emotional journey.

This review systematically examines the current state of research at the intersection of multimodal sensing, LLMs, and AER systems. The review will cover the definitions, methodologies, applications, effectiveness, system framework design, and limitations of multimodal sensing and LLM-driven AER systems, providing insights into them. The structure of this paper is as follows: Section 2 reviews related work and positions this review within the existing literature. Section 3 focuses on AER approaches, the status of MER, and LLM-enabled therapeutic dialogues. Section 4 offers AER architecture design and implementation and outlines its key challenges. Section 5 concludes this review.

## 2. Related Work

To effectively determine the contributions of this review, it is necessary to contextualize it within the existing landscape of scholarly surveys and position papers. The burgeoning research interest at the intersection of AI, sensing, and mental health has generated a body of literature, yet prior reviews have often examined the constituent components of AER systems in relative isolation. A logical progression from simpler, component-focused reviews to the need for a more integrated synthesis reveals the current gaps. Early work and subsequent reviews frequently concentrated on the application of general AI methods for mental health, such as analyzing biometric signals or passively sensed data for mental state detection or diagnostic support. Other lines of review have centered on the implementation and evaluation of specific digital therapeutic modalities, most notably digital CBT delivered via applications or basic chatbots, assessing their efficacy for conditions like insomnia or depression. Separately, the field of multimodal sensing has seen reviews dedicated to MER or mental state detection, meticulously detailing various modalities (e.g., facial expressions, voice acoustics, and physiological signals like ECG or EDA), data fusion techniques (such as early, late, or model-level fusion), and available datasets, though often without explicitly integrating these sensing capabilities into dynamic, LLM-driven therapeutic interaction frameworks [27,28]. More recently, the emergence of powerful LLMs has spurred reviews focused specifically on their capabilities, potential applications (like conversational agents or clinical support), credibility factors (reliability, explainability), and the significant ethical considerations surrounding their use in mental healthcare. However, even these recent LLM-focused reviews may not deeply integrate the complexities of multimodal sensing or the nuances of system design and user interaction specific to emotion recognition (ER) [29].

This tendency in the existing literature to examine components such as LLMs, multimodal sensing, specific therapeutic approaches (like CBT), system architectures, and user interaction dynamics in relative isolation results in a fragmented understanding. Such fragmentation hinders a holistic view of how these critical elements might synergistically combine to create truly effective and responsible AER. Furthermore, crucial aspects necessary for real-world viability are often underemphasized or treated peripherally within these narrower scopes. These include the complex dynamics of human–AI interaction within a sensitive therapeutic context [27,30], the design considerations for robust, scalable, and secure system frameworks [28,30,31], and the practical challenges associated with implementation, validation, and ER in clinical settings.

This review seeks to fill this identified gap by providing a unified perspective on the convergence of multimodal sensing and LLMs specifically for the development, understanding, and evaluation of AER systems. The primary aim is to explore the synergistic potential arising from combining advanced conversational AI with rich, multimodal sensing data while also critically examining the inherent technical, practical, and implementational challenges that emerge at their intersection. By synthesizing research across these previously fragmented areas, this review endeavors to offer a comprehensive resource for researchers, developers, clinicians, and policymakers navigating this complex and rapidly evolving field.

To further contextualize this review within the landscape of existing surveys and position papers, a detailed comparison highlighting the unique focus and scope of the present work is provided in Table 1. A mark ‘√’ indicates that the topic was addressed in the review, ‘Few’ indicates limited coverage, and ‘×’ indicates it was not discussed.

## 3. Automated Emotional Regulation: Approaches and Challenges

AER systems often work by digitizing established, evidence-based therapeutic approaches. These therapies are increasingly delivered via automated platforms, including websites, mobile apps, immersive virtual environments, and even physical robots.

A core challenge in replicating ER is accurately perceiving the patient’s emotional state. Human therapists naturally integrate various cues—observing facial expressions, listening to the tone of voice, and interpreting language—to understand a patient’s emotional state and tailor their approach. To emulate this crucial capability, AER systems are incorporating multimodal sensing technologies [45]. By capturing and analyzing data from visual (e.g., facial expressions), auditory (e.g., speech patterns and tone), and potentially physiological channels, these systems aim for a more accurate approach of ER. Achieving reliable recognition is vital, as understanding the user’s state is key to providing effective and responsive support.

**Figure 1:**
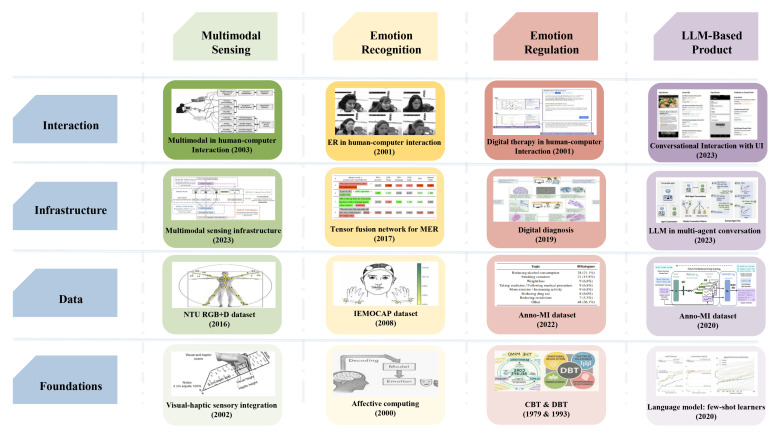
Milestones in multimodal sensing, emotion recognition, and LLM-based agents.

Beyond perception, delivering ER effectively requires interaction. While automating therapeutic dialogue has historically been difficult, the advent of LLMs offers new possibilities. LLM enable more fluent, context-aware, and potentially empathetic conversations compared to earlier chatbots, moving closer to the nuanced dialogue found in ER. Figure 1 illustrates key milestones in the development of multimodal emotion recognition and LLM-based agents, highlighting the convergence of these fields in recent years. A total of 16 key milestones are mapped onto a 4 × 4 matrix that spans four system layers—foundations, data, infrastructure, and interaction—and four converging research areas—multimodal sensing, ER, emotion regulation, and LLM-based products. The true potential of next-generation AER lies in bridging these rich multimodal inputs for perception with the advanced conversational capabilities of LLMs for interaction and delivery. This integration opens opportunities for interpreting complex human states more effectively and delivering highly personalized, context-aware therapeutic interventions.

Building on this foundation, this chapter provides an in-depth review of the entire process, from perceptual sensing techniques to emotional state intervention. Specifically, Section 3.1 evaluates the strengths and weaknesses of current unimodal sensing approaches and underscores the feasibility and potential advantages of adopting multimodal strategies within AER. Section 3.2 delves into the research on detecting emotional and emotional states using various sensing modalities. Finally, Section 3.3 discusses the advancements in developing LLM-based conversational agents tailored for emotional and emotional dialogue.

### 3.1. Status of Current Unimodal Emotional Recognition and Regulation Methods

AER systems often rely on first recognizing a user’s emotional state. Early research predominantly focused on unimodal approaches, analyzing single data streams like facial expressions, voice patterns, text sentiment, or physiological signals. These methods analyze isolated aspects of expression, seeking to infer emotions from visual cues, acoustic features, linguistic content, or bodily responses. While foundational, each unimodal strategy has distinct capabilities but also significant limitations when dealing with the complexity of human emotions in real-world settings. Table 2 provides an overview of the visual, auditory, physiological, and behavioral/contextual modalities employed in these systems, highlighting the trends and gaps in current implementations.

#### 3.1.1. Modalities for Automated Emotion Recognition

Automated emotion recognition systems typically integrate four complementary sensing modalities—visual, auditory, physiological, and behavioral/contextual—each capturing a distinct facet of the human affective response.

- Visual modality: Computer-vision algorithms applied to camera streams extract high-level representations of facial action units, eye-gaze trajectories, pupil dilation, and head– or body-movement kinematics. Large–scale, carefully curated videos [46,47,48] enable data-driven learning of robust visual affect representations.
- Auditory modality: Microphone recordings preserve rich affective cues embedded in speech acoustics—pitch, intensity, prosody, and temporal pause structure—as well as in the linguistic content of utterances. Because vocal signals are less susceptible to physical occlusion than faces, speech analysis supports non-intrusive, *always-on* monitoring. Modern natural-language-processing models further uncover the cognitive component of emotion conveyed in texts [49].
- Physiological modality: Wearable sensors (e.g., ECG/PPG watches, EDA bands, and EEG headsets) measure involuntary autonomic and central-nervous-system activity such as heart-rate variability, electrodermal conductance, respiration, and cortical rhythms. These signals provide continuous, high-reliability indices of arousal that are difficult to consciously manipulate [51]. Recent miniaturization of hardware has increased their feasibility in daily-life settings.
- Behavioral/contextual modality. Smartphones [52] and wearables [53] supply contextual traces—GPS trajectories, accelerometer-derived physical activity, communication logs, and device usage patterns—that situate other sensor streams within the user’s real-world environment, thereby enriching the interpretation of moment-to-moment affective states.

Taken together, these modalities furnish complementary views of emotion: vision excels at decoding facial expressions, audio at vocal nuance, language at explicit cognitive appraisal, physiology at latent arousal, and context at situational framing. This inherent diversity and the specific advantages of each modality directly inform how such systems should be built. Consequently, a recognition system hinges on selecting the modality set that best aligns with application objectives, privacy constraints, and deployment environments.

#### 3.1.2. Limitations of Unimodal Emotion Recognition Approaches

Although each sensing channel offers a distinctive vantage point on affect, unimodal systems fundamentally struggle to generalize effectively.

- **Vision** is vulnerable to occlusions (masks, glasses, and hand-to-face motion), extreme head poses, and adverse illumination. Facial expressions are also culturally moderated and can be deliberately suppressed or exaggerated, leading to systematic bias in both data and models [54]. Moreover, many everyday affective events lack a salient facial display, so purely visual systems often regress to guessing from sparse cues.
- **Audio** deteriorates rapidly under ambient noise, reverberation, packet loss, or low-quality microphones; even modest signal-to-noise degradation (<10 dB) can halve classification accuracy [55]. Speaker-dependent factors such as accent, age, health, and paralinguistic habits further confound counseling, while existing corpora over-represent acted emotions and read speech, thus limiting ecological validity.
- **Physiology** delivers involuntary signals, yet these traces are notoriously noisy: motion artifacts, electrode–skin impedance changes, temperature drift, and sensor displacement corrupt ECG/PPG and EDA. Inter-subject variability is large—baseline heart-rate variability can differ by a factor of two between healthy adults—necessitating cumbersome personal calibration. More critically, the mapping from raw autonomic activity to discrete affective states is many-to-many: elevated arousal may indicate fear, excitement, physical exertion, or caffeine intake, rendering interpretation context-dependent and ambiguous [56,57].
- **Behavioral/contextual** traces collected from smartphones and wearables (GPS, accelerometry, app usage, and communication logs) are typically sparse and irregular, dominated by missing data whenever devices are switched off, out of charge, or left behind. Location and activity cues are coarse-grained and can confound affect with routine (e.g., commuting) or exogenous factors (e.g., weather). Continuous tracking imposes non-trivial privacy, consent, and battery burdens, while interpretation is highly person- and culture-specific—the same mobility pattern may signal boredom in one user and relaxation in another. Finally, available datasets remain small and skewed toward convenience samples, hampering model generalization [58,59].

These modality-specific shortcomings—susceptibility to context, signal degradation, deliberate deception, and ambiguous affect mapping—collectively motivate multimodal fusion. By combining heterogeneous but complementary streams, a system can cross-validate inconsistent cues, fill information gaps, and exploit contextual priors, thereby achieving more robust, accurate, and ecologically valid ER than any unimodal pipeline in isolation.

#### 3.1.3. Multimodal Emotional Datasets

Based on the number of modalities involved, emotional corpora can be broadly categorized into bimodal and multimodal datasets. We summarize widely used bimodal and multimodal emotional databases in Table 3; categorized by the types of modalities, they include emotional and annotation formats, as well as application contexts.

These datasets typically adopt two dominant paradigms for emotional annotation: categorical labels and dimensional models. The categorical approach focuses on universal, discrete emotional states. These labels are widely used in facial expression datasets like AFEW [60] and RAVDESS [61].

In contrast, dimensional models, particularly the valence–arousal framework, conceptualize emotions as continuous states. Valence reflects the positive or negative quality of the emotion, while arousal indicates its intensity or energy level. This paradigm supports finer-grained analysis of emotional responses and is used in physiological datasets such as DEAP [62] and MAHNOB-HCI [63]. While dimensional models offer a representation of transient affective states, they still primarily capture momentary emotional responses rather than longer-term affective dispositions. Emotions are often short-lived reactions (e.g., anger or joy in response to a trigger), whereas moods are longer-term affective states (e.g., feeling gloomy or cheerful) not necessarily tied to a particular cause. This distinction is crucial for designing effective emotional regulation systems. Current emotion recognition datasets are predominantly emotion-oriented, focusing on immediate, observable affective cues.

Some datasets, such as IEMOCAP [64], include both categorical and dimensional annotations, providing a rich resource for evaluating diverse modeling strategies in multimodal emotion recognition systems.

**Table 3:** Overview of representative emotional datasets.

Dataset	Modalities	Emotion Labels	Participants/Size
eNTERFACE (2006) [65]	Visual and audio	Happiness, sadness, anger, fear, disgust, and surprise	1277 video clips
IEMOCAP (2008) [64]	Text, visual, audio, and body	Happiness, sadness, anger, frustration, neutral, and others; valence, dominance, and arousal	10,039 conversations
MAHNOB-HCI [63]	EEG, visual, audio, body, eye gaze, ECG, GSR, Temp, and Resp	Valence, arousal, dominance, and predictability; joy, amusement, sadness, disgust, anxiety, fear, surprise, anger, and neutral	27–30 participants; 20 clips per subject
AFEW (2012) [60]	Visual, audio	anger, disgust, fear, happiness, sadness, surprise, and neutral	1156 clips; 330 actors
DEAP (2012) [62]	EEG, EDA, ECG, EMG, EOG, RESP, BVP, SKT, visual, and audio	Valence, arousal, dominance, and liking	32 participants; 40 trials
CMU-MOSEI (2018) [66]	Text, visual, and audio	Happiness, sadness, anger, fear, disgust, and surprise	3229 video clips
RAVDESS (2018) [61]	Visual and audio	Neutral, calm, happy, sad, angry, fearful, disgust, and surprised	24 actors; 7356 files
MELD (2019) [67]	Text, visual, and audio	Anger, disgust, sadness, joy, neutral, surprise, and fear	1433 dialogues; 13,708 utterances
HEU (2021) [68]	Visual, audio, and body	Anger, boredom, confusion, disappointed, disgust, fear, happy, neutral, sad, and surprise	19,004 clips

### 3.2. Multimodal Sensing: Emotional State Perception and Recognition

Accurate perception of a patient’s emotional state is crucial for AER. However, traditional psychological assessment methods, such as clinical observation and patient self-reports, face considerable limitations in providing a consistently objective, continuous, and comprehensive understanding of a patient’s affective state. These methods can be susceptible to observer subjectivity, including issues like clinician bias and inter-rater variability, and patient reporting biases [69]. Furthermore, they often struggle to capture the dynamic, subtle, or non-verbalized nuances of emotional experiences and typically lack the granularity needed to map momentary affective changes that occur outside of structured assessment sessions. Multimodal sensing offers a technological solution, capturing data from multiple physiological and behavioral sources, including visual, auditory, and physiological signals, simultaneously [70,71]. Building upon the limitations of traditional self-report and clinician-based assessments, multimodal approaches have been proposed to improve emotional state perception and recognition. Table 4 summarizes the technical strategies across representative studies, including feature extraction, fusion methods, models used, and the types of emotional labels targeted.

#### 3.2.1. Signal Interpretation for Emotional Regulation

Transforming raw sensor data into therapeutically meaningful information requires processing and interpretation, heavily relying on machine learning (ML). First, raw signals undergo pre-processing and feature extraction (e.g., facial action units from video, Mel-frequency cepstral coefficients (MFCCs) from audio, statistical features from physiological signals, and location variance from GPS) to highlight relevant patterns. The core challenge is mapping these low-level features to high-level emotional labels in anxiety, engagement, and cognitive load. ML algorithms, including conventional methods and increasingly deep learning, such as CNNs [72], RNNs, LSTM [75], and transformers [53], learn these complex, often non-linear mappings. However, the “black box” nature of many deep learning (DL) models poses interpretability challenges, hindering clinical trust and adoption. Research in explainable AI and learning robust representations are crucial. Furthermore, sensor readings must be contextualized, as their meaning depends heavily on the situation, like high heart rates due to excitement vs. stress vs. exercise. Multimodal sensing aids contextualization by combining data streams, such as heart rate or accelerometers, and passive behavioral/environmental data.

#### 3.2.2. Affective Computing Techniques

Affective computing [78] develops systems to recognize, interpret, and simulate human emotions, underpinning multimodal emotional state perception in AER. It draws on emotional models (discrete categories like happiness/sadness or continuous dimensions like valence/arousal). A key focus is MER, integrating cues from facial expressions, voice, body language, and physiology for more robust and nuanced assessments compared to unimodal approaches. ML algorithms (SVMs, HMMs, and DL models like CNNs/RNNs) are essential for building these recognition systems. Multimodal fusion strategies, including feature-level, decision-level, and hybrid/model-level fusion, combine data streams.

#### 3.2.3. Emotion Recognition: Multimodal and Conversational Perspectives

MER is vital for AER systems to perceive and respond appropriately to user emotions, fostering effective therapeutic dialogue. This capability allows systems to adapt interactions based on the user’s detected state, enhancing personalization and potential effectiveness. Integrating ER, especially using multimodal approaches, into conversational AI is key to developing more emotionally intelligent and supportive mental health tools.

#### 3.2.4. Multimodal Emotion Recognition Pipeline

**Figure 2:**
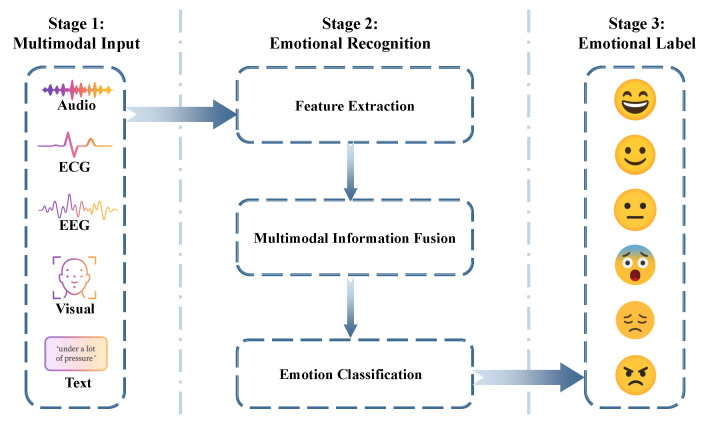
Three-stage multimodal emotion recognition pipeline.

Generally, MER is implemented by a three-stage pipeline as follows (Figure 2):

1. **Multimodal sensing** collects multimodal signal data and then subjects them to a feature extraction process.
2. **Multimodal information fusion** converts these raw streams into an integrated affect representation: modality-specific encoders first extract salient features; a fusion module then aligns and weights them before an emotion classifier infers the latent state.
3. **Emotional classification** maps the prediction to a discrete set of categories.

By feeding this label back to the dialogue manager at each turn, the system can modulate the language style, empathy level, and therapeutic strategy in real time. In this way, MER transforms passive sensing into an active conversational skill, enabling the AER agent to perceive, interpret, and respond to user emotions with a degree of personalization and emotional intelligence [79].

#### 3.2.5. Multimodal Fusion Strategies

Effectively integrating information from diverse modalities is central to MER. Various fusion strategies exist:

- Feature-level fusion combines extracted features from each modality into one vector before classification. This can capture low-level intermodal dependencies but is sensitive to missing data and requires feature alignment [76].
- Decision-level fusion trains separate classifiers per modality and combines their predictions later using methods like voting, averaging, or meta-classifiers. This is more robust to missing modalities but might miss subtle crossmodal interactions.
- Hybrid fusion combines aspects of both early and late fusion. Model-level fusion, which is common in deep learning, integrates representations learned at intermediate layers of modality-specific networks within a unified architecture. This allows adaptive learning of how to combine modalities. The choice of strategy impacts performance, robustness, and computational needs, which are especially crucial for real-time conversational systems where processing multiple streams is demanding [77].

#### 3.2.6. State-of-the-Art Performance in Multimodal Emotion Recognition

In addition to discussing the modalities and technical strategies of MER, it is essential to present a performance overview of state-of-the-art systems across both laboratory and in-the-wild settings. Table 5 summarizes representative studies that evaluate emotion recognition models on benchmark datasets using different combinations of modalities and models. These systems demonstrate strong performance in controlled lab settings, often exceeding 80% accuracy or F1 scores when using physiological or multimodal inputs. However, performance generally decreases in real-world conditions due to noise, occlusion, and ambiguous expressions. This shows the continuing challenge of building robust, generalizable emotion recognition systems that perform reliably outside the lab environment.

### 3.3. LLM-Enabled Dialogue-Based Therapist

Recent advancements in LLMs have significantly enhanced automated therapeutic interactions, especially models like ChatGPT, which have recognition due to their conversational capabilities, context awareness, and empathetic response generation. GPT-4 has demonstrated substantial improvements in nuanced understanding, contextual reasoning, and empathy simulation, making it highly effective for applications in automated emotional regulation.

In parallel, other emerging LLMs, such as DeepSeek and LLaMA2, have also entered the scene, each with distinctive strengths. DeepSeek has also shown its performance in fine-grained emotional understanding and customized dialogues, outperforming traditional models in certain emotional perception tasks due to its targeted fine-tuning on domain-specific datasets. Additionally, LLaMA2 excels in efficiency and scalability, enabling effective deployment even in resource-constrained environments. These LLMs models provide valuable alternatives or supplements for building more adaptive AER systems.

Table 6 outlines key studies employing LLM-based strategies for emotional regulation, categorizing them by technique, LLM type, and therapy focus.

#### 3.3.1. LLM Capabilities for Therapeutic Interaction

LLMs possess capabilities that are promising for therapeutic contexts. Their advanced natural language understanding and generation enable fluent, coherent, and context-aware conversations, surpassing traditional chatbots. While lacking genuine emotions, LLMs can simulate empathy through learned language patterns, generating validating and supportive responses that are often perceived as non-judgmental by users. This can be enhanced using techniques based on therapeutic principles, such as Chain-of-Empathy prompting. LLMs can be guided via prompting or fine-tuning to adhere to evidence-based ER protocols, delivering techniques such as cognitive restructuring or Socratic questioning in a structured yet conversational manner. They offer potential for personalization, adapting responses based on user input and history. Furthermore, techniques like retrieval-augmented generation (RAG) [92] allow LLMs to integrate external, vetted knowledge, potentially improving response accuracy and reliability.

However, such empathic language generation does not equate to true empathy, which requires affective understanding and intention, a faculty LLMs fundamentally lack. Clinical evaluations have cautioned that LLM responses, while linguistically appropriate, may fail to detect nuanced emotional states or respond safely in high-risk scenarios, such as self-harm or trauma disclosure [89,93,94]. Therefore, claims regarding the therapeutic capability of LLMs should be tempered and framed as “perceived empathy” under controlled settings rather than authentic affective engagement.

#### 3.3.2. Current Applications

LLMs are being applied across various mental health domains. Conversational agents, like chatbots, powered by LLMs aim to provide accessible support and psychoeducation, as well as deliver therapies like CBT, showing potential for symptom reduction and engagement [86,88]. However, their safety and efficacy require rigorous evaluation. Different strategies like zero/few-shot prompting [87], fine-tuning [90], and RAG [91] are used, with fine-tuning often yielding higher accuracy but requiring more resources. LLMs can also assist clinicians by summarizing notes or providing decision support, thereby optimizing workflows. In research, they generate synthetic data, simulate users for chatbot evaluation, and aid qualitative data analysis.

### 3.4. Overview of Available Multimodal LLM-Based AER Systems

While the previous sections focused on individual techniques and modalities, this section highlights several integrated AER systems that combine multimodal sensing with large language models. Table 7 summarizes representative systems, outlining the types of sensory inputs, LLM technologies employed, therapeutic grounding, and deployment methods. These systems reflect the growing interest in creating real-world emotional support tools, but most remain in early-stage development or limited evaluation.

## 4. Insight and Key Challenges

Following the discussion presented in Section 3.3, multimodal perception integrated with an LLM-based therapist emerges as a promising approach for developing advanced AER systems. In this chapter, we first examine the availability and effectiveness of such multimodal integration. Subsequently, we highlight current challenges and propose potential directions for future research.

### 4.1. Automated Emotional Regulation Architecture Design

**Figure 3:**
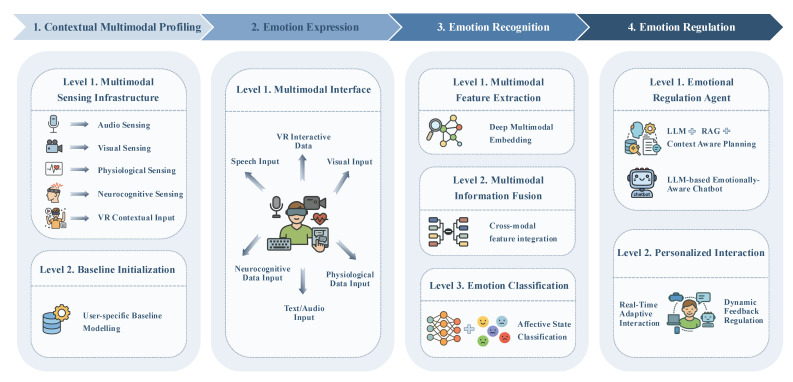
Framework of the proposed multimodal sensing LLM design.

Multimodal sensing-enabled LLM-based AER systems are anticipated to significantly enhance automated therapeutic interactions by leveraging multimodal data integration and the advanced contextual understanding capabilities of LLMs. To achieve this vision, two critical aspects must be addressed: (1) defining an appropriate multimodal architecture that optimizes the integration and utilization of diverse sensory inputs and (2) establishing methods to equip therapeutic agents with the capability to effectively interpret and respond to emotional states. In terms of architectural design, we systematically synthesize existing multimodal sensing and information fusion techniques, culminating in our proposed unified framework (see Figure 3) comprising four integral modules: contextual multimodal profiling (CMP), emotion expression, ER, and emotion regulation. Concerning capability enhancement, we categorize the strategies based on their reliance on fine-tuning LLM or incorporating RAG. In subsequent sections, we delve deeper into the details and implications of our proposed multimodal therapeutic framework.

#### 4.1.1. Contextual Multimodal Profiling Module

CMP supplies the raw yet context-aware substrate that every subsequent module, especially the ER block, builds upon.

- Personal profile ingestion: Parallel to baseline recording, CMP collects the following static personal information via a privacy-preserving wizard: age, gender identity, language, cultural background, clinically relevant history, and medication. Structured answers populate a secure profile store, while data are summarized by an on-device database into key–value pairs.
- Multimodal sensing infrastructure: A network of co-located microphones, RGB-D/eye-tracking cameras, wearable ECG/EDA/EEG devices, and VR/IoT loggers continuously streams audio–visual, physiological, neurocognitive, and interaction data in lock-step with textual inputs captured by the LLM interface. Each packet carries a high-precision Unix timestamp to guarantee sub-10 ms alignment across channels, while an adaptive sampling controller throttles or boosts individual sensors according to battery, bandwidth, and privacy constraints.
- Baseline initialization: During onboarding the user performs neutral tasks for a few minutes. Gaussian-process priors model trait physiology, habitual vocal prosody, and typical postural ranges; live signals are thereafter z-normalized against these priors so that only state-driven deviations remain salient.

#### 4.1.2. Emotion Expression Module

The emotion expression module translates the regulation agent’s abstract intentions into observable, emotionally aligned behavior across the channels the user actually perceives. At each conversation with the chatbot, the LLM receives the user’s recognized affect vector plus high-level therapeutic goals. Prompt templates employing Chain-of-Empathy and evidence-based counseling schemas guide the model to choose an expression strategy—validating reflection, gentle cognitive re-appraisal, motivational challenge, or silent presence. A crossmodal synchronizer keeps audio, visual, and haptic frames together, and the selected strategies are mapped onto coordinated output modalities.

By fusing LLM-driven empathy with synchronised multimodal rendering, the EEM turns therapeutic intent into felt support, closing the perception–action loop that underpins automated emotion regulation.

#### 4.1.3. Emotion Recognition Module

The ER module converts the calibrated, context-rich streams delivered by CMP into a compact affect vector that can be queried at every dialogue turn. It follows the classical three-stage multimodal ER pipeline—feature extraction, information fusion, and emotion classification—which has been widely adopted in affective computing systems.

- Feature extraction: Synchronously sampled audio, visual, textual, physiological, and neurocognitive channels are first routed through lightweight modality-specific encoders.
- Information fusion: To handle signal drop-outs and exploit crossmodal synergies, a hybrid strategy is adopted. A model-level transformer aligns the embeddings through crossmodal attention so co-occurring cues—e.g., elevated pitch and increased EDA—reinforce each other, while a decision-level ensemble remains available as a fallback option when an entire modality disappears. This combination inherits the robustness of late fusion and the sensitivity of early fusion, which together have the potential to address the trade-offs discussed in recent MER studies.

Emotion classification: A multi-task head projects the fused representation onto (i) continuous valence–arousal coordinates, (ii) six basic-emotion logits, and (iii) an epistemic-uncertainty scalar that downstream safety layers can query.

#### 4.1.4. Emotion Regulation Module

**Figure 4:**
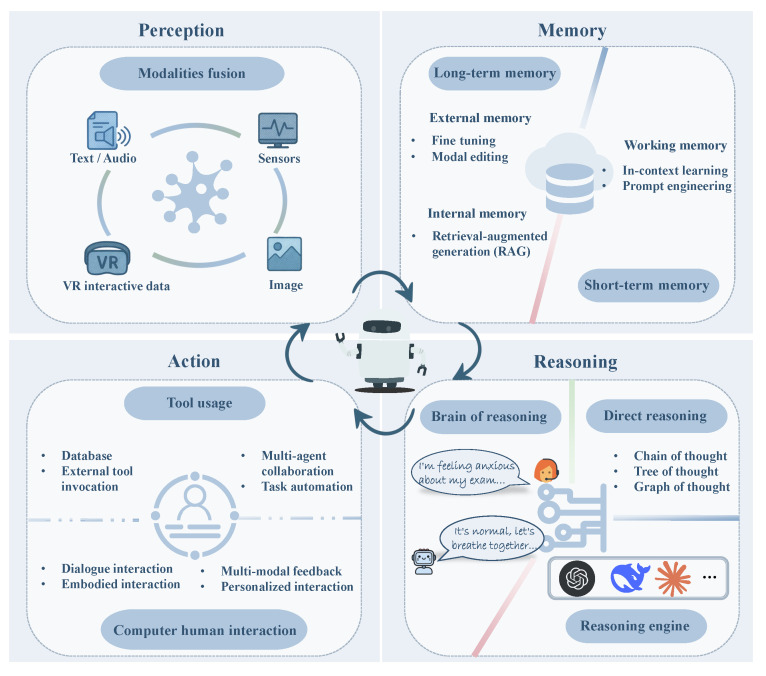
Emotional regulation cycle: perception, memory, reasoning, and action.

The Emotion regulation module is engineered to translate therapeutic intent into context-sensitive actions. This is conceptualized as a continuous closed-loop cycle, as illustrated in Figure 4, encompassing perception, memory, reasoning, and action, which collectively enable the system to sense, interpret, decide, and respond adaptively to user emotions.

The cycle initiates with perception, where diverse multimodal data streams, including texts, speech, images, biometrics, and interactive data from VR environments, are fused to create a moment-to-moment snapshot of the user’s affective state. This snapshot is then processed by the memory component, which stores it in short-term buffers while simultaneously integrating it with long-term, fine-tuned knowledge via RAG. This rich, blended context forms the foundation for the reasoning stage.

The reasoning process is primarily orchestrated by what can be termed the regulation agent layer, a multi-agent chatbot system. Within this layer, a planner first decomposes the current therapeutic goal into more granular, fine-grained subtasks. Concurrently, a real-time emotion-adaptive dialogue controller injects the latest affect vector, derived from the perception stage, into the system’s prompt. These two signals—the decomposed therapeutic subtask and the live affect vector—form a joint query for a retriever. This retriever performs RAG over a vetted corpus of therapeutic scripts, such as those from CBT, DBT, and mindfulness practices, ranking passages whose regulation techniques best fit the specific goal–state pair. These clinically relevant, grounded excerpts are then fed back to the LLM. This enables the LLM to select clinically appropriate regulation strategies, leveraging mechanisms such as chain-, tree-, or graph-of-thought strategies, and to dynamically modulate its tone, verbosity, and output modalities while remaining clinically aligned.

Subsequently, the chosen strategy is operationalized in the action phase through what is realized as the HCI layer. This layer translates the selected strategy into tangible outputs through tool invocations, multi-agent collaboration, and embodied interactions. The specific intervention is rendered through a device-adaptive Webot interface, ensuring a coherent expression of a single therapeutic plan across various channels via a shared multimodal API. These channels include (i) A mobile or web application featuring a conversational chatbox, micro-learning cards, and biometric-triggered nudges. (ii) A VR environment where an embodied avatar conducts interventions such as paced-breathing exercises, exposure scenes, or group-skills practice, often with synchronised audio–haptics. (iii) A physical robot, whether tabletop or humanoid, which can use gaze, gestures, and the physical hand-over of small objects to facilitate behavioral rehearsal.

Finally, the resulting feedback from these user interactions re-enters the perception stage. This crucial step closes the loop, allowing the system to continuously update its understanding and maintain an adaptive alignment, ensuring it remains responsive to the user’s evolving emotional state.

### 4.2. Implementation and Methodological Insights

To bridge the gap between conceptual design and practical deployment, we provides a detailed account of the data processing pipeline, model architecture, and therapeutic integration strategies employed in multimodal sensing-enabled LLM systems.

First, raw signals collected from heterogeneous sensing modalities, including physiological, visual, auditory, and neurocognitive inputs, undergo pre-processing to reduce noise, enhance signal quality, and ensure temporal alignment. Standard techniques such as bandpass filtering for ECG and PPG, low-pass filtering and decomposition for EDA, and artifact removal for EEG are applied to physiological signals [100]. Audio and visual data are preprocessed using conventional denoising methods and feature-enhancing pipelines, such as MFCC extraction for speech [101] and facial landmark detection for video. All sensor streams are synchronized through high-resolution timestamps to enable reliable downstream multimodal fusion and real-time processing.

Then, feature extraction and multimodal fusion are conducted using a combination of conventional and deep learning approaches. Each modality is processed through a dedicated encoder, such as CNNs for visual input or transformer-based models for sequential data, to derive representative features [102]. These features are then integrated via a hybrid fusion strategy, combining model-level attention-based fusion with a decision-level fallback ensemble to enhance robustness in the presence of noisy or missing data. The resulting affective representation typically includes continuous valence, arousal estimates, and discrete emotion categories, serving as the input for subsequent emotional reasoning and response generation.

Third, the emotion regulation component leverages LLM to generate context-aware therapeutic responses based on the user’s emotional state and interaction history. Prompt templates are designed to embed both affective cues and therapeutic objectives, drawing on frameworks such as CBT approaches. Where necessary, lightweight fine-tuning, such as LoRA [103] and RAG, are used to improve outputs.

### 4.3. Key Challenges

The development of multimodal, LLM-driven AER systems represents a significant frontier in mental health technology, yet it is fraught with profound challenges that span the entire architectural stack, from data acquisition to therapeutic intervention. These challenges are not merely technical hurdles but are deeply intertwined with fundamental issues in psychology, clinical safety, and ethics. This section deconstructs these challenges by mapping them onto the core modules of the AER architecture: CMP, emotion expression, ER, and emotion regulation, as well as overarching systemic issues.

#### 4.3.1. Challenges in Contextual Multimodal Profiling

The challenges in CMP are foundational, as failures at this initial data acquisition stage can invalidate the entire system. A primary obstacle is maintaining data fidelity in uncontrolled real-world settings. Visual and audio data are often compromised by variable illumination, occlusions, and background noise, while wearable sensors are susceptible to low signal-to-noise ratios from non-emotional factors like physical activity [104]. A more fundamental issue is the “observer effect,” where the act of being monitored alters a user’s emotional state, leading them to mask or suppress feelings [105].

Besides, emotion is an inherently subjective experience shaped by culture and context, leading to low inter-annotator agreement—sometimes below 10% consensus [106]. Forcing complex, mixed emotions into discrete labels is a gross oversimplification that trains models on an incomplete representation of reality, leading critics to argue that emotion recognition verges on pseudoscience built on a tenuous foundation.

To address these challenges, we propose the integration of federated learning frameworks [107] to decentralize data training and preserve user privacy without exposing raw data. Moreover, embedding differential privacy mechanisms and on-device learning can further mitigate risks associated with continuous sensing.

#### 4.3.2. Challenges in Emotion Expression

The emotion expression module, the user-facing component of the AER system, must overcome significant HCI challenges to build trust and rapport. An immense technical challenge is achieving multimodal behavioral synchronization, replicating the tightly coordinated dance of speech, intonation, gaze, and gesture found in human communication [108]. The agent’s non-verbal behaviors must be synchronized both temporally and semantically with the LLM’s linguistic output, a complex task that requires bridging the semantic-motor gap between high-level text and low-level motor commands [109].

Second, the agent must project not just empathy, but authentic empathy. Simulated empathy that feels hollow or formulaic can be perceived as manipulative and damage user trust. True empathy requires real-time responsiveness to the user’s cues, necessitating a tight, closed-loop integration with the system’s perception modules to create a genuinely adaptive and contingent interaction. Besides, cultural variability in emotional expression and interpretation poses an additional challenge. For example, in some cultures, a gentle smile during emotionally intense conversations may signal empathy and composure, while in others it could be misinterpreted as emotional distance or even insincerity. Similarly, emotional restraint is often valued in some location areas contexts but may be mistaken for disengagement by models trained predominantly on Western datasets [110]. Therefore, AER systems must incorporate culturally adaptive expression strategies to ensure alignment with diverse user expectations and norms.

A feasible approach to enhance the authenticity of empathy is reinforcement learning with human feedback (RLHF) [111], where human evaluators rate the perceived empathy of agent responses. This feedback loop fine-tunes expression strategies toward more organically adaptive and emotionally aligned behaviors. Additionally, dynamic empathy regulation can be achieved by grounding output modulation in real-time affect recognition, creating a tighter perception–action loop.

#### 4.3.3. Challenges in Emotion Recognition

The ER module, which interprets the incoming data, faces algorithmic and representational hurdles. A core challenge is the robust fusion of multimodal information, as there is no single best method, and choices involve trade-offs between strategies like early, late, and hybrid fusion [104]. These methods must also handle missing or low-quality data, as a single noisy stream can poison the entire process, and many models degrade severely if one modality is unavailable.

Similarly, creating subject-independent models that work for new users without extensive personalization is a major challenge due to significant individual differences in emotional expression [112]. Addressing this requires advanced techniques like domain adaptation and domain generalization, which are themselves complex in a multimodal context. Finally, the real-time computational feasibility of these systems is a major barrier. State-of-the-art ER models are computationally intensive, leading to high latency that is unsuitable for in-the-moment regulation. Deploying these large models on resource-constrained hardware like smartphones and wearables creates a difficult trade-off between model accuracy and efficiency, a critical issue in a high-stakes domain like mental health.

To address the above challenges in emotion recognition, several strategies can be adopted. First, robust multimodal fusion can be enhanced by employing adaptive fusion architectures, such as transformer based models with modality-specific attention, capable of weighting informative signals while suppressing noisy channels. For missing data, modality dropout training and imputation techniques like conditional variational autoencoders can maintain performance. Second, to improve generalizability, domain-adaptive learning frameworks, e.g., adversarial domain adaptation or meta-learning, can help transfer learned features across users and contexts.

#### 4.3.4. Challenges in Emotion Regulation

The emotion regulation module, the therapeutic core of the system, confronts high-stakes challenges in clinical safety, ethics, and long-term user impact. Ensuring the clinical validity and safety of the LLM’s outputs is paramount, as these models are prone to hallucinations, generating plausible but clinically inappropriate or harmful advice [113,114,115]. To mitigate this, LLM responses must be rigorously grounded in evidence-based therapeutic protocols like CBT, often using techniques like RAG to constrain outputs to a trusted knowledge base.

Moreover, crisis detection and escalation remains underdeveloped in many current AER systems. Instances where AI fails to adequately respond to disclosures of self-harm or suicidal ideation raise significant ethical and practical red flags [116]. Effective systems must incorporate robust crisis escalation protocols, including (1) real-time detection of high-risk intent using multimodal sentiment and keyword analysis, (2) automatic redirection to human support channels, (3) and fallback safety mechanisms that override automated responses when necessary. These systems must be designed with fail-safe redundancy to ensure timely intervention and avoid harm. The challenge of defining accountability for such decisions, particularly when harm arises from AI-generated advice, remains an ongoing area of ethical inquiry.

To further strengthen system trustworthiness, bias mitigation strategies [117] should be employed throughout the model development pipeline. These include the use of diverse training datasets, the application of fairness-aware learning algorithms, and the auditing of system performance across cultural, gender, and age groups to prevent unequal treatment or misinterpretation of emotional states.

#### 4.3.5. Overarching Systemic and Ethical Challenges

Beyond the individual modules, the AER system faces overarching systemic and ethical challenges that require a holistic perspective. The continuous collection of highly sensitive biometric and personal data raises profound privacy, security, and consent issues. Implementing consent-driven data architectures, encrypted storage, and user-controllable data governance is essential.

Architecturally, integrating the distinct CMP, emotion expression, ER, and emotion regulation modules into a cohesive, low-latency system is a formidable engineering task. This modularity, while technically advantageous, can also face the problem of fragmented architecture of AER systems, comprising separate modules for sensing, interpretation, and responses. The ‘responsibility gap’ complicates ethical evaluation and risk management.

Finally, the field suffers from a lack of standardized evaluation benchmarks for validating the clinical safety and efficacy of AI-driven mental health interventions. While new frameworks and datasets are emerging, the absence of standards makes it difficult for developers, regulators, and users to assess, compare, and trust these novel and powerful systems.

A comprehensive governance framework should be established, drawing from existing standards such as the FDA SaMD framework [118] and ISO/IEC 27001 [119]. Key components include the (1) consent-aware logging architecture, (2) user-controlled data dashboards for access and deletion, and (3) third-party algorithmic audits for bias and safety. Additionally, ethics review boards and continuous post-deployment monitoring should be mandated for any clinical-grade AER system. Standardized evaluation metrics, e.g., clinical outcomes, emotional alignment, and fairness scores, should be developed to enable transparent comparison and benchmarking of AER system efficacy and safety.

## 5. Conclusions

The integration of multimodal sensing technologies and LLMs for AER systems presents a field with significant potential but also considerable complexities. The convergence of these technologies marks a shift towards more improved AER, moving beyond basic systems to those capable of understanding nuanced emotional states through diverse data streams—physiological, behavioral, and contextual. LLMs, with their advanced empathetic response generation and language understanding, are key to interpreting this data and offering tailored support. This opens opportunities for highly personalized, adaptive, and proactive AER tools applicable in mental healthcare, education, workplace well-being, and human-computer interaction, potentially revolutionizing access to emotional support.

However, these advancements are accompanied by substantial technical challenges. Technical hurdles include the need for high-quality multimodal datasets; the complexity of integrated models, ensuring generalizability; and the limitations of current sensing technologies. Ethically, the continuous monitoring of personal emotional data raises concerns about privacy, data security, algorithmic bias, potential over-reliance on these systems, and the risk of misuse, compounded by the opacity of many AI models.

The field of multimodal LLM-based AER is still in its early stages, especially regarding real-world deployment and validated long-term efficacy. Future progress must prioritize safety, ethical integrity, and clinical utility. Key next steps include developing robust data governance frameworks, researching bias mitigation, advancing model interpretability, establishing standardized evaluation methodologies for efficacy and safety, and fostering a system where technology augments human agency. Ultimately, the development of AER systems must adopt a socio-technical perspective, recognizing their deep embeddedness within human social and psychological landscapes. The promise of enhancing well-being through these technologies can only be realized via a collective commitment to responsible innovation, rigorous validation, and steadfast adherence to ethical principles, ensuring that advanced emotional support genuinely empowers individuals.

## Figures and Tables

**Table 1:** Comparisons between other related reviews and our paper (√ = covered; Few = partially; × = not addressed).

Publication	Multimodal Sensing	Emotion Expression	Emotion Recognition	Emotion Regulation	AI Methods (Non-LLM)	LLMs	User Interaction	Discussion of Framework Design
Sun et al., 2023 [30]	√	√	√	√	√	√	×	×
Khoo et al., 2024 [27]	√	√	√	Few	√	×	×	×
Lutz et al., 2022 [28]	√	×	×	√	Few	×	Few	×
Bendig et al., 2022 [32]	×	√	√	√	√	×	Few	×
Xu et al., 2022 [31]	×	√	√	√	√	×	√	√
Lim et al., 2022 [33]	×	√	Few	√	×	×	√	×
Olawade et al., 2024 [34]	Few	√	√	√	√	×	√	×
Zhou et al., 2022 [35]	×	Few	√	Few	√	×	√	×
Ray et al., 2022 [36]	Few	Few	×	√	√	×	√	×
Boucher et al., 2021 [37]	×	Few	Few	√	√	×	√	√
Gual-Montolio et al., 2022 [38]	×	Few	Few	√	√	×	√	×
Thomas et al., 2007 [39]	×	×	×	√	×	×	√	√
D’Alfonso1 et al., 2017 [40]	×	×	×	√	√	×	√	√
Pham et al., 2022 [41]	×	×	×	×	√	×	√	√
Kourtesis et al., 2024 [42]	Few	×	×	×	√	×	√	√
Xiao et al., 2025 [43]	×	√	×	√	√	√	√	√
Wang et al., 2024 [44]	Few	Few	√	×	×	√	×	Few
Guo et al., 2024 [29]	Few	×	×	Few	√	√	√	×
Our Paper	√	√	√	√	√	√	√	√

**Table 2:** Overview of sensing modalities used across reviewed AER systems (√ = covered; Few = partially; × = not addressed).

Publication	Visual	Auditory	Physiological	Behavioral/Contextual
Middya et al., 2022 [46]	√	√	×	×
Noroozi et al., 2017 [47]	√	√	×	×
Tzirakis et al., 2017 [48]	√	√	×	×
Zhang et al., 2024 [49]	√	√	×	×
Zhao et al., 2021 [50]	√	√	√	×
Pantic et al., 2003 [51]	√	√	√	×
Kang et al., 2023 [52]	×	√	×	√
Wu et al., 2023 [53]	×	×	√	√

**Table 4:** Approaches to multimodal emotional state perception and recognition in reviewed studies.

Publication	Feature Extraction	Fusion Method	Classifier/Model	Emotion Labels
Al et al., 2019 [72]	Electrodermal activity	×	CNN (with grid search tuning)	Discrete
Younis et al., 2022 [73]	On-body physiological markers	Feature fusion	KNN, DT, RF, and SVM + DT meta	Discrete
Hossain et al., 2019 [74]	Electrodermal activity	×	CNN (with grid search tuning)	Discrete
Alhagry et al., 2017 [75]	ERaw EEG signals	×	LSTM + Dense Layer	Valence–Arousal + Liking
Wu et al., 2023 [53]	Multimodal physiological signals (ECG, EDA, EMG, and Resp)	Transformer-based shared encoder	SSL with modality-specific Conv transformer encoders	Discrete
Cai et al., 2020 [76]	Linear & non-linear EEG features	Feature-level fusion	KNN, DT, and SVM	Binary (Depressed/Control)
Cimtay et al., 2020 [77]	Facial expressions, GSR, and EEG	Hybrid fusion	CNN-based + fusion layers	Discrete

**Table 5:** Performance of representative ER systems in lab and wild environments.

Publication	Dataset	Model	Environment	Accuracy/F1
Tomar et al., 2024 [80]	eNTERFACE	Multimodal (feature-level fusion and CNN + MLP)	Lab	62.93%
Tripathi et al., 2018 [81]	IEMOCAP	Multimodal contextual LSTM with 3D-CNN, Text-CNN, and openSMILE	Lab	71.59%
Wiem et al., 2017 [82]	MAHNOB-HCI	SVM (with Gaussian kernel best)	Lab	Arousal: 64.23% and valence: 68.75%
Kossaifi et al., 2017 [83]	AFEW	MKL (shape + DCT)	In-the-wild	CORR = 0.445 and ICC = 0.340
Tripathi et al., 2017 [84]	DEAP	CNN	Lab	Valence (2-class): 81.41% Arousal (2-class): 73.36% Valence (3-class): 66.79% Arousal (3-class): 57.58%
Zadeh Tripathi et al., 2018 [66]	CMU-MOSEI	Graph-MFN	Wild	Sentiment: Acc = 76.9 and F1 = 77.0 Emotion (happy): WA = 69.1 and F1 = 76.6 Emotion (sad): WA = 66.3 and F1 = 66.3
Luna-Jimenez et al., 2021 [85]	RAVDESS	Fine-tuned CNN-14 (PANNs) + STN + bi-LSTM + late fusion	Lab	80.08%
Chen et al., 2021 [68]	HEU	MMA (face + speech + body)	Wild	Val: 55.04%

**Table 6:** Overview of emotional regulation techniques enabled by LLMs and related technologies.

Publication	Technique	LLM Involvement	Therapy Type
Wu et al., 2023 [86]	LLM-based multi-agent collaboration	GPT-4	Potential for CBT-style management
Wang et al., 2024 [87]	LLM-based prompting	GPT-3.5/GPT-4	Supportive emotional co-regulation
Chiu et al., 2024 [88]	LLM-based chatbot	ChatGPT and Flama	Supportive conversation
Qiu et al., 2024 [89]	Zero-shot prompting	GPT-4	Supportive conversation and counselor–client interactions
Na et al., 2024 [90]	Zero-shot prompting and fine-tuned LLMs using structured CBT prompts and QA dataset	GPT-3.5-turbo	Derived from CBT and the CBT dataset
Antico et al., 2024 [91]	Contextualized informational support via a RAG-enhanced chatbot	GPT-4	Supportive conversation

**Table 7:** Overview of available AER systems integrating multimodal sensing and LLM technologies.

Publication	Modalities	LLMs	Therapy Type	Annotations
Chu et al., 2024 [95]	Text, audio, and video	BlenderBot, GPT-3.5, GPT-4.0, and video-LLaMA	Emotional support therapy; AI-based conversational agent	Applied/ research prototype
Yang et al., 2024 [96]	Text, image, audio, and video	LLaMA2-7B and GPT-4V	Emotional understanding	Theoretical/ research prototype
Zhang et al., 2025 [97]	EEG, text, audio, and wearable sensor	GPT-3.5, GPT-4.0, and video-LLaMA	Evidence-based intervention	Applied/ research prototype
Laban et al., 2025 [98]	Audio, text, and video	GPT-3.5	Cognitive reappraisal; robot in university	Applied/ product-level
Dong et al., 2024 [99]	Audio, text, and video	Baichuan-13B-chat	Psychological profiling; digital psychological; avatar multimodal	Hybrid/ research prototype

## References

[1] Carbonell A., Navarro-Pérez J.J., Mestre M.V. (2020). Challenges and barriers in mental healthcare systems and their impact on the family: A systematic integrative review. Health Soc. Care Community.

[2] Wienand D., Wijnen L.I., Heilig D., Wippel C., Arango C., Knudsen G.M., Goodwin G.M., Simon J. (2024). Comorbid physical health burden of serious mental health disorders in 32 European countries. BMJ Ment. Health.

[3] Solmi M., Radua J., Olivola M., Croce E., Soardo L., Salazar de Pablo G., Il Shin J., Kirkbride J.B., Jones P., Kim J.H. (2022). Age at onset of mental disorders worldwide: Large-scale meta-analysis of 192 epidemiological studies. Mol. Psychiatry.

[4] World Health Organization Mental Disorders. https://www.who.int/news-room/fact-sheets/detail/mental-disorders.

[5] Walker E.R., McGee R.E., Druss B.G. (2015). Mortality in mental disorders and global disease burden implications: a systematic review and meta-analysis. JAMA Psychiatry.

[6] Brouwers E.P. (2020). Social stigma is an underestimated contributing factor to unemployment in people with mental illness or mental health issues: Position paper and future directions. BMC Psychol..

[7] Weizenbaum J. (1966). ELIZA—A computer program for the study of natural language communication between man and machine. Commun. ACM.

[8] Fitzpatrick K.K., Darcy A., Vierhile M. (2017). Delivering cognitive behavior therapy to young adults with symptoms of depression and anxiety using a fully automated conversational agent (Woebot): A randomized controlled trial. JMIR Ment. Health.

[9] Elyoseph Z., Hadar-Shoval D., Asraf K., Lvovsky M. (2023). ChatGPT outperforms humans in emotional awareness evaluations. Front. Psychol..

[10] Martinengo L., Stona A.C., Griva K., Dazzan P., Pariante C.M., von Wangenheim F., Car J. (2021). Self-guided cognitive behavioral therapy apps for depression: systematic assessment of features, functionality, and congruence with evidence. J. Med. Internet Res..

[11] Clarke J., Proudfoot J., Whitton A., Birch M.R., Boyd M., Parker G., Manicavasagar V., Hadzi-Pavlovic D., Fogarty A. (2016). Therapeutic alliance with a fully automated mobile phone and web-based intervention: Secondary analysis of a randomized controlled trial. JMIR Ment. Health.

[12] Klein B., Meyer D., Austin D.W., Kyrios M. (2011). Anxiety online—A virtual clinic: Preliminary outcomes following completion of five fully automated treatment programs for anxiety disorders and symptoms. J. Med. Internet Res..

[13] Freeman D., Lister R., Waite F., Yu L.M., Slater M., Dunn G., Clark D. (2019). Automated psychological therapy using virtual reality (VR) for patients with persecutory delusions: Study protocol for a single-blind parallel-group randomised controlled trial (THRIVE). Trials.

[14] Lambe S., Knight I., Kabir T., West J., Patel R., Lister R., Rosebrock L., Rovira A., Garnish B., Freeman J. (2020). Developing an automated VR cognitive treatment for psychosis: gameChange VR therapy. J. Behav. Cogn. Ther..

[15] Bălan O., Moldoveanu A., Leordeanu M. (2021). A machine learning approach to automatic phobia therapy with virtual reality. Modern Approaches to Augmentation of Brain Function.

[16] Barua P.D., Vicnesh J., Gururajan R., Oh S.L., Palmer E., Azizan M.M., Kadri N.A., Acharya U.R. (2022). Artificial intelligence enabled personalised assistive tools to enhance education of children with neurodevelopmental disorders—A review. Int. J. Environ. Res. Public Health.

[17] Garcia-Ceja E., Riegler M., Nordgreen T., Jakobsen P., Oedegaard K.J., Tørresen J. (2018). Mental health monitoring with multimodal sensing and machine learning: A survey. Pervasive Mob. Comput..

[18] Zhou D., Luo J., Silenzio V., Zhou Y., Hu J., Currier G., Kautz H. Tackling mental health by integrating unobtrusive multimodal sensing. Proceedings of the AAAI Conference on Artificial Intelligence.

[19] Xu X., Yao B., Dong Y., Gabriel S., Yu H., Hendler J., Ghassemi M., Dey A.K., Wang D. (2024). Mental-llm: Leveraging large language models for mental health prediction via online text data. Proc. ACM Interactive Mobile Wearable Ubiquitous Technol..

[20] Li C., Wang J., Zhang Y., Zhu K., Hou W., Lian J., Luo F., Yang Q., Xie X. (2023). Large language models understand and can be enhanced by emotional stimuli. arXiv.

[21] Alvarez-Gonzalez N., Kaltenbrunner A., Gómez V. (2021). Uncovering the limits of text-based emotion detection. arXiv.

[22] Kim B.H., Wang C. (2025). Large Language Models for Interpretable Mental Health Diagnosis. arXiv.

[23] Adhikary P.K., Srivastava A., Kumar S., Singh S.M., Manuja P., Gopinath J.K., Krishnan V., Gupta S.K., Deb K.S., Chakraborty T. (2024). Exploring the efficacy of large language models in summarizing mental health counseling sessions: Benchmark study. JMIR Ment. Health.

[24] Cabrera J., Loyola M.S., Magaña I., Rojas R. (2023). Ethical dilemmas, mental health, artificial intelligence, and llm-based chatbots. Proceedings of the International Work-Conference on Bioinformatics and Biomedical Engineering.

[25] Yuan A., Garcia Colato E., Pescosolido B., Song H., Samtani S. (2025). Improving workplace well-being in modern organizations: A review of large language model-based mental health chatbots. ACM Trans. Manag. Inf. Syst..

[26] Yu H., McGuinness S. (2024). An experimental study of integrating fine-tuned LLMs and prompts for enhancing mental health support chatbot system. J. Med. Artif. Intell..

[27] Khoo L.S., Lim M.K., Chong C.Y., McNaney R. (2024). Machine learning for multimodal mental health detection: A systematic review of passive sensing approaches. Sensors.

[28] Lutz W., Schwartz B., Delgadillo J. (2022). Measurement-based and data-informed psychological therapy. Annu. Rev. Clin. Psychol..

[29] Guo Z., Lai A., Thygesen J.H., Farrington J., Keen T., Li K. (2024). Large language models for mental health applications: Systematic review. JMIR Ment. Health.

[30] Sun J., Dong Q.X., Wang S.W., Zheng Y.B., Liu X.X., Lu T.S., Yuan K., Shi J., Hu B., Lu L. (2023). Artificial intelligence in psychiatry research, diagnosis, and therapy. Asian J. Psychiatry.

[31] Xu B., Zhuang Z. (2022). Survey on psychotherapy chatbots. Concurr. Comput. Pract. Exp..

[32] Bendig E., Erb B., Schulze-Thuesing L., Baumeister H. (2022). The next generation: Chatbots in clinical psychology and psychotherapy to foster mental health—A scoping review. Verhaltenstherapie.

[33] Lim S.M., Shiau C.W.C., Cheng L.J., Lau Y. (2022). Chatbot-delivered psychotherapy for adults with depressive and anxiety symptoms: A systematic review and meta-regression. Behav. Ther..

[34] Olawade D.B., Wada O.Z., Odetayo A., David-Olawade A.C., Asaolu F., Eberhardt J. (2024). Enhancing mental health with Artificial Intelligence: Current trends and future prospects. J. Med. Surgery Public Health.

[35] Zhou S., Zhao J., Zhang L. (2022). Application of artificial intelligence on psychological interventions and diagnosis: An overview. Front. Psychiatry.

[36] Ray A., Bhardwaj A., Malik Y.K., Singh S., Gupta R. (2022). Artificial intelligence and Psychiatry: An overview. Asian J. Psychiatry.

[37] Boucher E.M., Harake N.R., Ward H.E., Stoeckl S.E., Vargas J., Minkel J., Parks A.C., Zilca R. (2021). Artificially intelligent chatbots in digital mental health interventions: A review. Expert Rev. Med. Devices.

[38] Gual-Montolio P., Jaén I., Martínez-Borba V., Castilla D., Suso-Ribera C. (2022). Using artificial intelligence to enhance ongoing psychological interventions for emotional problems in real-or close to real-time: A systematic review. Int. J. Environ. Res. Public Health.

[39] Thomas R., Zimmer-Gembeck M.J. (2007). Behavioral outcomes of parent-child interaction therapy and Triple P—Positive Parenting Program: A review and meta-analysis. J. Abnorm. Child Psychol..

[40] D’alfonso S., Santesteban-Echarri O., Rice S., Wadley G., Lederman R., Miles C., Gleeson J., Alvarez-Jimenez M. (2017). Artificial intelligence-assisted online social therapy for youth mental health. Front. Psychol..

[41] Pham K.T., Nabizadeh A., Selek S. (2022). Artificial intelligence and chatbots in psychiatry. Psychiatr. Q..

[42] Kourtesis P. (2024). A Comprehensive Review of Multimodal XR Applications, Risks, and Ethical Challenges in the Metaverse. Multimodal Technol. Interact..

[43] Xiao H., Zhou F., Liu X., Liu T., Li Z., Liu X., Huang X. (2025). A comprehensive survey of large language models and multimodal large language models in medicine. Inf. Fusion.

[44] Wang J., Jiang H., Liu Y., Ma C., Zhang X., Pan Y., Liu M., Gu P., Xia S., Li W. (2024). A comprehensive review of multimodal large language models: Performance and challenges across different tasks. arXiv.

[45] Wang P., Liu A., Sun X. (2025). Integrating emotion dynamics in mental health: A trimodal framework combining ecological momentary assessment, physiological measurements, and speech emotion recognition. Interdiscip. Med..

[46] Middya A.I., Nag B., Roy S. (2022). Deep learning based multimodal emotion recognition using model-level fusion of audio–visual modalities. Knowl.-Based Syst..

[47] Noroozi F., Marjanovic M., Njegus A., Escalera S., Anbarjafari G. (2017). Audio-visual emotion recognition in video clips. IEEE Trans. Affect. Comput..

[48] Tzirakis P., Trigeorgis G., Nicolaou M.A., Schuller B.W., Zafeiriou S. (2017). End-to-end multimodal emotion recognition using deep neural networks. IEEE J. Sel. Top. Signal Process..

[49] Zhang S., Yang Y., Chen C., Zhang X., Leng Q., Zhao X. (2024). Deep learning-based multimodal emotion recognition from audio, visual, and text modalities: A systematic review of recent advancements and future prospects. Expert Syst. Appl..

[50] Zhao S., Jia G., Yang J., Ding G., Keutzer K. (2021). Emotion recognition from multiple modalities: Fundamentals and methodologies. IEEE Signal Process. Mag..

[51] Pantic M., Rothkrantz L.J. (2003). Toward an affect-sensitive multimodal human-computer interaction. Proc. IEEE.

[52] Kang S., Choi W., Park C.Y., Cha N., Kim A., Khandoker A.H., Hadjileontiadis L., Kim H., Jeong Y., Lee U. (2023). K-emophone: A mobile and wearable dataset with in-situ emotion, stress, and attention labels. Sci. Data.

[53] Wu Y., Daoudi M., Amad A. (2023). Transformer-based self-supervised multimodal representation learning for wearable emotion recognition. IEEE Trans. Affect. Comput..

[54] Li S., Deng W., Du J. (2017). Reliable Crowdsourcing and Deep Locality-Preserving Learning for Expression Recognition in the Wild. Proceedings of the 2017 IEEE Conference on Computer Vision and Pattern Recognition (CVPR).

[55] Schuller B.W., Batliner A., Bergler C., Messner E.M., Hamilton A., Amiriparian S., Baird A., Rizos G., Schmitt M., Stappen L. (2020). The Interspeech 2020 Computational Paralinguistics Challenge: Elderly Emotion, Breathing & Masks. https://www.isca-archive.org/interspeech_2020/schuller20_interspeech.html.

[56] Pané-Farré C.A., Alius M.G., Modeß C., Methling K., Blumenthal T., Hamm A.O. (2015). Anxiety sensitivity and expectation of arousal differentially affect the respiratory response to caffeine. Psychopharmacology.

[57] Raz S., Lahad M. (2022). Physiological indicators of emotional arousal related to ANS activity in response to associative cards for psychotherapeutic PTSD treatment. Front. Psychiatry.

[58] Harari Y., Shawen N., Mummidisetty C.K., Albert M.V., Kording K.P., Jayaraman A. (2021). A smartphone-based online system for fall detection with alert notifications and contextual information of real-life falls. J. Neuroeng. Rehabil..

[59] Chan S., Santoro A., Lampinen A., Wang J., Singh A., Richemond P., McClelland J., Hill F. (2022). Data distributional properties drive emergent in-context learning in transformers. Adv. Neural Inf. Process. Syst..

[60] Dhall A., Goecke R., Lucey S., Gedeon T. (2012). Collecting Large, Richly Annotated Facial-Expression Databases from Movies. IEEE MultiMedia.

[61] Livingstone S.R., Russo F.A. (2018). The Ryerson Audio-Visual Database of Emotional Speech and Song (RAVDESS): A dynamic, multimodal set of facial and vocal expressions in North American English. PLoS ONE.

[62] Koelstra S., Muhl C., Soleymani M., Lee J., Yazdani A., Patras I. (2012). DEAP: A Database for Emotion Analysis; Using Physiological Signals. IEEE Trans. Affect. Comput..

[63] Soleymani M., Lichtenauer J., Pun T., Pantic M. (2011). A Multimodal Database for Affect Recognition and Implicit Tagging. IEEE Trans. Affect. Comput..

[64] Busso C., Bulut M., Lee C.C., Kazemzadeh A., Mower E., Kim S., Chang J.N., Lee S., Narayanan S.S. (2008). IEMOCAP: Interactive emotional dyadic motion capture database. Lang. Resour. Eval..

[65] Martin O., Kotsia I., Macq B., Pitas I. The eNTERFACE’05 Audio-Visual Emotion Database. Proceedings of the First IEEE Workshop on Multimedia Database Management (ICDEW’06).

[66] Zadeh A.B., Liang P.P., Poria S., Cambria E., Morency L.P. Multimodal language analysis in the wild: Cmu-mosei dataset and interpretable dynamic fusion graph. Proceedings of the 56th Annual Meeting of the Association for Computational Linguistics (Volume 1: Long Papers).

[67] Poria S., Hazarika D., Majumder N., Naik G., Cambria E., Mihalcea R. MELD: A Multimodal Multi-Party Dataset for Emotion Recognition in Conversation. Proceedings of the 57th Annual Meeting of the Association for Computational Linguistics.

[68] Chen J., Wang C., Wang K., Yin C., Zhao C., Xu T., Zhang X., Huang Z., Liu M., Yang T. (2021). HEU Emotion: A Large–Scale Database for Multi–modal Emotion Recognition in the Wild. Neural Comput. Appl..

[69] García J.A.B. (2011). Description and limitations of instruments for the assessment of expressed emotion. Papeles Psicólogo.

[70] Guo R., Guo H., Wang L., Chen M., Yang D., Li B. (2024). Development and application of emotion recognition technology—A systematic literature review. BMC Psychol..

[71] Pillalamarri R., Shanmugam U. (2025). A review on EEG-based multimodal learning for emotion recognition. Artif. Intell. Rev..

[72] Al Machot F., Elmachot A., Ali M., Al Machot E., Kyamakya K. (2019). A deep-learning model for subject-independent human emotion recognition using electrodermal activity sensors. Sensors.

[73] Younis E.M., Zaki S.M., Kanjo E., Houssein E.H. (2022). Evaluating ensemble learning methods for multi-modal emotion recognition using sensor data fusion. Sensors.

[74] Hossain M.S., Muhammad G. (2019). Emotion recognition using deep learning approach from audio–visual emotional big data. Inf. Fusion.

[75] Alhagry S., Fahmy A.A., El-Khoribi R.A. (2017). Emotion recognition based on EEG using LSTM recurrent neural network. Int. J. Adv. Comput. Sci. Appl..

[76] Cai H., Qu Z., Li Z., Zhang Y., Hu X., Hu B. (2020). Feature-level fusion approaches based on multimodal EEG data for depression recognition. Inf. Fusion.

[77] Cimtay Y., Ekmekcioglu E., Caglar-Ozhan S. (2020). Cross-subject multimodal emotion recognition based on hybrid fusion. IEEE Access.

[78] Picard R.W. (2000). Affective Computing.

[79] Chow K., Fritz T., Holsti L., Barbic S., McGrenere J. (2023). Feeling stressed and unproductive? A field evaluation of a therapy-inspired digital intervention for knowledge workers. ACM Trans. Comput.-Hum. Interact..

[80] Tomar P.S., Mathur K., Suman U. (2024). Fusing facial and speech cues for enhanced multimodal emotion recognition. Int. J. Inf. Technol..

[81] Tripathi S., Beigi H. (2018). Multi-modal emotion recognition on IEMOCAP with neural networks. arXiv.

[82] Wiem M.B.H., Lachiri Z. (2017). Emotion classification in arousal valence model using MAHNOB-HCI database. Int. J. Adv. Comput. Sci. Appl..

[83] Kossaifi J., Tzimiropoulos G., Todorovic S., Pantic M. (2017). AFEW-VA database for valence and arousal estimation in-the-wild. Image Vis. Comput..

[84] Tripathi S., Acharya S., Sharma R., Mittal S., Bhattacharya S. Using deep and convolutional neural networks for accurate emotion classification on DEAP data. Proceedings of the AAAI Conference on Artificial Intelligence.

[85] Luna-Jiménez C., Griol D., Callejas Z., Kleinlein R., Montero J.M., Fernández-Martínez F. (2021). Multimodal emotion recognition on RAVDESS dataset using transfer learning. Sensors.

[86] Wu Q., Bansal G., Zhang J., Wu Y., Li B., Zhu E., Jiang L., Zhang X., Zhang S., Liu J. (2023). Autogen: Enabling next-gen llm applications via multi-agent conversation. arXiv.

[87] Wang J., Xiao Y., Li Y., Song C., Xu C., Tan C., Li W. (2024). Towards a client-centered assessment of llm therapists by client simulation. arXiv.

[88] Chiu Y.Y., Sharma A., Lin I.W., Althoff T. (2024). A computational framework for behavioral assessment of llm therapists. arXiv.

[89] Qiu H., Lan Z. (2024). Interactive agents: Simulating counselor-client psychological counseling via role-playing llm-to-llm interactions. arXiv.

[90] Na H. (2024). CBT-LLM: A Chinese large language model for cognitive behavioral therapy-based mental health question answering. arXiv.

[91] Antico C., Giordano S., Koyuturk C., Ognibene D. (2024). Unimib Assistant: Designing a student-friendly RAG-based chatbot for all their needs. arXiv.

[92] Lewis P., Perez E., Piktus A., Petroni F., Karpukhin V., Goyal N., Küttler H., Lewis M., Yih W.t., Rocktäschel T. (2020). Retrieval-augmented generation for knowledge-intensive nlp tasks. Adv. Neural Inf. Process. Syst..

[93] Sorin V., Brin D., Barash Y., Konen E., Charney A., Nadkarni G., Klang E. (2024). Large language models and empathy: Systematic review. J. Med. Internet Res..

[94] Sanjeewa R., Iyer R., Apputhurai P., Wickramasinghe N., Meyer D. (2024). Empathic conversational agent platform designs and their evaluation in the context of mental health: Systematic review. JMIR Ment. Health.

[95] Chu Y., Liao L., Zhou Z., Ngo C.W., Hong R. (2024). Towards multimodal emotional support conversation systems. arXiv.

[96] Yang Q., Ye M., Du B. (2024). Emollm: Multimodal emotional understanding meets large language models. arXiv.

[97] Zhang S., Hu Y., Yi X., Nanayakkara S., Chen X. (2025). IntervEEG-LLM: Exploring EEG-Based Multimodal Data for Customized Mental Health Interventions. Companion Proceedings of the ACM on Web Conference 2025.

[98] Laban G., Wang J., Gunes H. (2025). A Robot-Led Intervention for Emotion Regulation: From Expression to Reappraisal. arXiv.

[99] Dong T., Liu F., Wang X., Jiang Y., Zhang X., Sun X. (2024). Emoada: A multimodal emotion interaction and psychological adaptation system. Proceedings of the International Conference on Multimedia Modeling.

[100] Aqajari S.A.H., Naeini E.K., Mehrabadi M.A., Labbaf S., Dutt N., Rahmani A.M. (2021). pyeda: An open-source python toolkit for pre-processing and feature extraction of electrodermal activity. Procedia Comput. Sci..

[101] Ittichaichareon C., Suksri S., Yingthawornsuk T. Speech recognition using MFCC. Proceedings of the International Conference on Computer Graphics, Simulation and Modeling.

[102] Shaikh M.B., Chai D., Islam S.M.S., Akhtar N. (2024). From CNNs to transformers in multimodal human action recognition: A survey. ACM Trans. Multimed. Comput. Commun. Appl..

[103] Peng L., Zhang Z., Pang T., Han J., Zhao H., Chen H., Schuller B.W. (2024). Customising general large language models for specialised emotion recognition tasks. Proceedings of the ICASSP 2024—2024 IEEE International Conference on Acoustics, Speech and Signal Processing (ICASSP).

[104] Zhang Q., Wei Y., Han Z., Fu H., Peng X., Deng C., Hu Q., Xu C., Wen J., Hu D. (2024). Multimodal fusion on low-quality data: A comprehensive survey. arXiv.

[105] Udahemuka G., Djouani K., Kurien A.M. (2024). Multimodal Emotion Recognition using visual, vocal and Physiological Signals: A review. Appl. Sci..

[106] Plaza-del Arco F.M., Curry A., Curry A.C., Hovy D. (2024). Emotion analysis in NLP: Trends, gaps and roadmap for future directions. arXiv.

[107] Huang W., Wang D., Ouyang X., Wan J., Liu J., Li T. (2024). Multimodal federated learning: Concept, methods, applications and future directions. Inf. Fusion.

[108] Louwerse M.M., Dale R., Bard E.G., Jeuniaux P. (2012). Behavior matching in multimodal communication is synchronized. Cogn. Sci..

[109] Lee Y.K., Jung Y., Kang G., Hahn S. (2023). Developing social robots with empathetic non-verbal cues using large language models. arXiv.

[110] Mesquita B. (2001). Culture and emotion: Different approaches to the question. Emotions: Currrent Issues and Future Directions.

[111] Liu G.K.M. (2023). Transforming Human Interactions with AI via Reinforcement Learning with Human Feedback (RLHF).

[112] Ali M., Al Machot F., Haj Mosa A., Jdeed M., Al Machot E., Kyamakya K. (2018). A globally generalized emotion recognition system involving different physiological signals. Sensors.

[113] Goodman K.E., Paul H.Y., Morgan D.J. (2024). AI-generated clinical summaries require more than accuracy. JAMA.

[114] Rydzewski N.R., Dinakaran D., Zhao S.G., Ruppin E., Turkbey B., Citrin D.E., Patel K.R. (2024). Comparative evaluation of LLMs in clinical oncology. NEJM AI.

[115] Bedi S., Liu Y., Orr-Ewing L., Dash D., Koyejo S., Callahan A., Fries J.A., Wornow M., Swaminathan A., Lehmann L.S. (2024). Testing and evaluation of health care applications of large language models: A systematic review. JAMA.

[116] Qiu H., Zhao T., Li A., Zhang S., He H., Lan Z. (2023). A benchmark for understanding dialogue safety in mental health support. Proceedings of the CCF International Conference on Natural Language Processing and Chinese Computing.

[117] Dai S., Xu C., Xu S., Pang L., Dong Z., Xu J. Bias and unfairness in information retrieval systems: New challenges in the llm era. Proceedings of the 30th ACM SIGKDD Conference on Knowledge Discovery and Data Mining.

[118] U.S. Food and Drug Administration (2024). Software as a Medical Device (SaMD). https://www.fda.gov/medical-devices/digital-health-center-excellence/software-medical-device-samd.

[119] (2022). Information Security, Cybersecurity and Privacy Protection—Information Security Management Systems—Requirements.

